# Scoliosis may be the result or cause of pelvic obliquity in Duchenne muscular dystrophy

**DOI:** 10.1590/1806-9282.20242070

**Published:** 2025-07-07

**Authors:** Josef Finsterer, Carla Alexandra Scorza, Fulvio Alexandre Scorza

**Affiliations:** 1 Neurology and Neurophysiology Center, Department of Neurology – Vienna, Austria.; 2 Universidade Federal de São Paulo, Escola Paulista de Medicina – São Paulo (SP), Brazil.

Dear Editor,

We were interested to read the article by Uğur et al. on a cross-sectional study of the relationship between pelvic obliquity and trunk control, spinal deformity, functional upper limb performance, and motor function in 21 non-ambulatory patients with Duchenne muscular dystrophy (DMD)^1^. Pelvic obliquity correlated with anterior spinal deformity, motor function, duration of wheelchair use, and knee flexion contractures^1^. It was concluded that pelvic tilt can lead to worsening spinal condition and impaired motor function in non-ambulatory DMD patients, especially those with lower extremity joint contractures and long-term wheelchair use^1^. The study is excellent, but some ambiguities should be clarified.

The first point is that motor function in DMD patients may depend on cardiac function^2^. Therefore, it would have been important to include cardiac function in the assessment. How many of the included patients had dilated cardiomyopathy, chronic heart failure, reduced ejection fraction or fractional shortening, or elevated pro-brain natriuretic peptide (pro-BNP)? How many of the included patients were on regular heart failure therapy? Motor function in DMD may also depend on lung function, as adequate oxygenation is a prerequisite for normal muscle function^3^. How many of the 21 included patients had impaired lung function, in particular, how many required oxygen supplementation? How many were on non-invasive ventilation?

The second point is that the scoliosis in DMD can be so severe that heart and lung functions are impaired. In such cases, surgical stabilization of the spine is performed to improve cardiac and pulmonary function^4^. How many of the included patients had previously undergone spinal stabilization surgery?

The third point is that motor function in DMD is highly dependent on the underlying genetic defect^5^. Was there a correlation between the type and amount of mutation in the dystrophin gene in the included patients? Was the pelvic obliquity associated with the deletion of certain exons in the dystrophin gene?

The fourth point is that pelvic obliquity may also depend on the degree of muscle weakness of the pelvic girdle muscles. Was the strength of the pelvic girdle muscles measured, and was there a correlation between the degree of pelvic tilt and the asymmetric muscle weakness of the pelvic girdle muscles?

The fifth point is that pelvic obliquity can be the cause of scoliosis, but it should be kept in mind that scoliosis can also cause pelvic obliquity^6^. How could it be excluded that the scoliosis caused the pelvic obliquity, and not vice versa, i.e., the pelvic obliquity caused the scoliosis in the included patients?

To summarize, this interesting study has limitations that put the results and their interpretation into perspective. Removing these limitations could strengthen the conclusions and reinforce the message of the study. All unanswered questions need to be clarified before readers can uncritically accept the conclusions of the study. Pelvic obliquity in DMD can cause scoliosis, but conversely, scoliosis can also cause pelvic obliquity.

## Data Availability

The datasets generated and/or analyzed during the current study are available from the corresponding author upon reasonable request.
